# TrkB-Target Galectin-1 Impairs Immune Activation and Radiation Responses in Neuroblastoma: Implications for Tumour Therapy

**DOI:** 10.3390/ijms19030718

**Published:** 2018-03-02

**Authors:** Katharina Batzke, Gabriele Büchel, Wiebke Hansen, Alexander Schramm

**Affiliations:** 1 Department of Medical Oncology, West German Cancer Center, University Hospital Essen, University of Duisburg-Essen, 45122 Essen, Germany; katharina.batzke@uk-essen.de; 2 Theodor Boveri Institute and Comprehensive Cancer Center Mainfranken, Biocenter, University of Würzburg, Am Hubland, 97074 Würzburg, Germany; gabriele.buechel@uni-wuerzburg.de; 3 Institute of Medical Microbiology, University Hospital Essen, University of Duisburg-Essen, 45122 Essen, Germany; wiebke.hansen@uk-essen.de

**Keywords:** Galectin-1, radiation response, neuroblastoma

## Abstract

Galectin-1 (Gal-1) has been described to promote tumour growth by inducing angiogenesis and to contribute to the tumour immune escape. We had previously identified up-regulation of Gal-1 in preclinical models of aggressive neuroblastoma (NB), the most common extracranial tumour of childhood. While Gal-1 did not confer a survival advantage in the absence of exogenous stressors, Gal-1 contributed to enhanced cell migratory and invasive properties. Here, we review these findings and extend them by analyzing Gal-1 mediated effects on immune cell regulation and radiation resistance. In line with previous results, cell autonomous effects as well as paracrine functions contribute to Gal-1 mediated pro-tumourigenic functions. Interfering with Gal-1 functions in vivo will add to a better understanding of the role of the Gal-1 axis in the complex tumour-host interaction during immune-, chemo- and radiotherapy of neuroblastoma.

## 1. Biology of Galectins—Physiology and Pathophysiology

Glycan-binding proteins were first described in the 1960s by Ashwell and Morrell [1], and this paved the way for identifying the family of Galectins. Galectins are highly conserved in the animal kingdom and differ from other lectins by their affinity for β-galactoside sugars. To date, 15 different Galectins have been found in mammals, but still many biological functions of these proteins are unknown [2,3]. They share a consensus carbohydrate recognition domain (CRD) comprising approximately 130 amino acids. This domain determines affinity for β-galactosides and allows for formation of galectin-glycan lattices. Based on CRD organisation, Galectins can be classified into three different groups: Galectin-1 is a member of the prototypical group, which is characterised by a single CRD that links monomers to form multimeric Galectin lattices. A second group, encompassing Galectin-4 and -5, among others, contains tandem-repeats with two non-identical CRDs connected by an amino acid linker. A third type of Galectins is referred to as “chimaera-type”, because the CRD is here fused to proline- and glycine-rich stretches. The only member of this group found to date is galectin-3 [2,4]. Galectins are mainly cytosolic proteins, but they are also localised in the extracellular space while lacking a typical secretion signal peptide. Thus, Galectins are secreted by a non-classical and yet poorly understood mechanism [5,6].

As a member of the prototypical group, Galectin-1 (Gal-1) is divalent and its ability to cross-link carbohydrate chains on cell surfaces has been recognised early on [4]. Gal-1 is a multifunctional protein involved in development and differentiation. It also plays a role in cell-cell adhesion and cell-matrix interaction and thus regulates intercellular communication processes [3]. However, Gal-1 is dispensable for normal development in mice, as Gal-1^−/−^ mice are healthy and fertile. Interestingly, targeted infection of Gal-1^−/−^ mice results in severe autoimmune disease, pointing to a role of Gal-1 in regulating and terminating inflammatory responses. This process has been attributed to increased Th1 and Th17 responses in Gal-1^−/−^ mice. Interestingly, Th2 cells…seemed to be protected from the anti-proliferative effects of Gal-1 as a consequence of different glycosylation patterns [7]. It has been discussed that Gal-1 is also critical for regulating inflammation-mediated neurodegeneration in experimental autoimmune encephalomyelitis (EAE), which is the murine equivalent to multiple sclerosis (MS). This hypothesis was supported by the observation that progressive neurodegeneration occurs, when Gal-1 is depleted in the acute phase of EAE, while adoptive transfer of Gal1-secreting astrocytes or administration of recombinant Gal-1 suppressed EAE [8]. Thus, Gal-1 seems to exert a critical role especially in regulating neuroinflammatory processes.

Another important physiological function of Gal-1 has been described in mediating feto-maternal tolerance [4]. It has been suggested that suppression of maternal immune responses against placental alloantigens and pregnancy maintenance are both affected by Gal-1. Interestingly, decreased Gal-1 levels were observed in women suffering a miscarriage [9]. Similarly, preeclampsia, a pregnancy-specific disorder characterized by sudden onset of hypertension and proteinuria, seems to be correlated with Gal-1 levels [10]. Taken together, those findings contribute to the notion that Gal-1 is required for healthy gestation.

In tumours, mainly the intracellular functions of Gal-1 have been linked to pro-tumourigenic process, as Gal-1 can interact with oncogenic H-RAS to promote angiogenesis [11], while Galectin 3 (Gal-3) and K-RAS proteins are found to team up in an unholy alliance in cancer cells. These findings seem to apply to many K-RAS driven malignancies including pancreatic cancer [12] and colon tumours [13]. Here, Gal-3 has been identified as an amplifier of K-RAS signalling via PI-3 kinase [14], which is in part mediated by Gal-3 facilitated formation of K-RAS nanoclusters [15]. Similarly, Gal-1 dimers were reported to scaffold Raf-effectors to increase H-RAS nanoclusters [16,17]. This conformation regulates the magnitude of RAS-driven MAPK-signal output [18]. While these functions refer to intracellular activity of Galectins, Gal-1 is also a paracrine mediator of tumour aggressiveness by acting as a pro-angiogenic factor [19,20]. Paracrine functions of Gal-3 include up-regulation of IL-6 in the tumour microenvironment via Gal-3BP (Galectin-3 binding protein), which promotes angiogenesis and inflammation [21]. However, Gal-3 can also exert autocrine effects in neuroblastoma cells by increasing phenotypic differentiation and by impairing MYCN-primed apoptosis via modulation of HIPK2 [22,23,24]. As both, Gal-1 and Gal-3, are highly expressed in the majority of neuroblastoma, it is necessary to better understand their interplay in regulating neuroblastoma proliferation and interaction with the tumour microenvironment.

**Figure 1:**
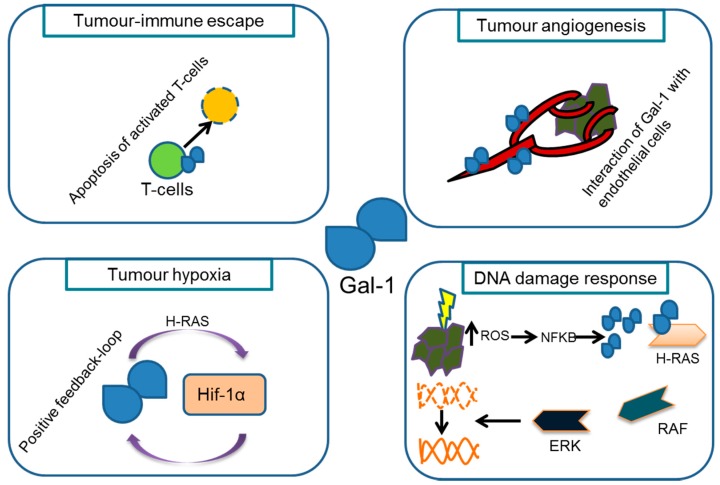
Schematic illustration of the multiple functions of Gal-1 in tumor-host interactions. Gal-1 contributes to the tumour-immune escape and promotes tumor angiogenesis by stimulating endothelial cell proliferation. Furthermore, Gal-1 is induced by (tumour) hypoxia and could contribute to radioresistance in hypoxic conditions by enabling a H-RAS dependent positive feedback-loop, which in turn induces Hif1α. Gal-1 was also reported to modulate DNA damage responses through activation of ROS and the H-RAS-RAF pathway (modified from [28]).

However, accumulating evidence suggest that both Gal-1 is linked to many hallmarks of cancer (Figure 1) and that the expression of Gal-1 correlates with tumour aggressiveness in different human cancer types including oral squamous cell carcinoma, ovarian and breast cancer [25]. Several strategies have thus been proposed to interfere with Gal-1 functions, including neutralising peptides [20], small molecule inhibitors, and supply with metabolic inhibitors of N-acetyllactosamine biosynthesis [26,27]. Several clinical trials evaluate safety and efficacy of inhibiting Gal-3 functions, mostly in combination with immune therapies. On the other hand, it still poses a major challenge to translate current knowledge into the design and development of effective galectin-1 inhibitors in cancer therapy.

## 2. Gal-1 Expression in Neuroblastoma Is Linked to TrkB Activation

Neuroblastoma (NB) is the most common extracranial solid tumour of childhood and arises from the sympathetic nervous system [29]. Interestingly, neuroblastoma is characterized by a wide variety of clinical outcomes ranging from spontaneous regression to rapid progression and aggressive growth in other patients. Clinical observations and molecular analyses led to a classification of NB into two types according to patient outcome, cytogenetics and gene and protein expression profiles [29]. The type 1 neuroblastoma is the non-aggressive, favourable subtype, presenting with a hyperploid karyotype and expression of the TrkA neurotrophin receptor. By contrast, type 2 NB are characterised by a diploid karyotype with structural aberrations including loss of chromosome 1p and amplification of the MYCN oncogene [30]. Type 2 NB also highly express the receptor tyrosine kinase TrkB, but not TrkA. In normal tissue, activation of TrkB by its ligand BDNF (brain-derived neurotrophic factor) triggers neuronal development and survival, but also mediates synaptic reorganization processes through different signalling cascades involving protein-protein interaction [31]. There are three main pathways induced upon TrkB activation. First, PLC-γ activation causes release of inositol-1,4,5-triphosphat (IP3) and diacylglycerol (DAG) to increase intracellular calcium concentrations, which in turn activate Ca^2+^-dependent protein kinases to stimulate cell growth. Secondly, TrkB induces the PI-3 kinase signalling pathway to activate AKT. Additionally, induction of the MAP/ERK pathway downstream of Ras/B-Raf promotes differentiation or cell survival depending on signal strength and duration [31,32]. The latter seems to be important for mediating the different effects exerted by the structurally highly related neurotrophin receptors, TrkA and TrkB. Analyses of the subtle differences between both receptors is deemed crucial for understanding the divergent biological outcomes of neurotrophin signalling in different cell types.

To further understand the biological consequences of the differently expressed neurotrophin receptors, TrkA and TrkB, we previously selected a Trk-negative human neuroblastoma cell line, SH-SY5Y, and designed subclones with either high TrkA or TrkB expression. Analysis of transcriptome and proteome changes revealed that Gal-1 is up-regulated only in SY5Y cells with ectopic TrkB expression (SY5Y-TrkB) [33,34]. As stimulation of TrkB by its ligand, BDNF, increases cell proliferation and resistance to chemotherapy in SY5Y-TrkB cells [35], we hypothesized that Gal-1 could contribute to TrkB mediated aggressiveness. This was supported by RNA profiling analysis of primary NB, which revealed a strong positive correlation between the expression of Gal-1 and TrkB, while expression of TrkA and Gal-1 were anti-correlated. Moreover, activation of TrkA or TrkB by their specific ligands in vitro confirmed up-regulation of Gal-1 only upon BDNF-mediated TrkB stimulation [36]. BDNF-mediated invasiveness of SY5Y-TrkB cells could be significantly reduced by neutralizing Gal-1 function, while recombinant Gal-1 could not fully recover the invasive phenotype of SY5Y-TrkB cells in the absence of BDNF. We concluded that Gal-1 is an important but not the only effector of BDNF-induced invasiveness of aggressive neuroblastoma cells [36].

## 3. Gal-1 Gene Dose Alters the Immune Phenotype and Tumour Angiogenesis in a Mouse Model of Neuroblastoma

Several mouse models have been developed to study neuroblastoma development in vivo as a consequence of MYCN or ALK overexpression in neural crest cell progenitors. While recent models used Cre-Lox technology to induce MYCN or ALK [37,38], an extensively characterized model has been developed by Weiss et al. more than 20 years ago. These transgenic mice (TH-MYCN) express the MYCN oncogene under control of the Tyrosine Hydroxylase (TH) promotor. They develop neuroblastic tumours comparable to those seen in humans concerning localisation, additional genomic aberrations and histological characteristics [39]. To investigate the function and biological role of Gal-1 in vivo, a Gal-1^−/−^ mouse strain was generated by Poirier and co-workers back in 1993. They used homologous recombination techniques in embryonic stem cells to disrupt and inactivate the L14 lectin gene (the original name of the mouse homologue for LGALS1, the Gal-1 encoding gene). Animals with a global knock-out of Gal-1 were indistinguishable from control littermates. The mice were fertile and developed normally with an unimpaired lifespan indicating that other highly related lectins may be able to compensate for the function of Gal-1 in these animals [40]. As pointed out above, Gal-1 is crucial for maintaining feto-maternal tolerance in allogeneic matings, but did not affect survival of new-born mice from syngeneic breeding [41]. Impaired tolerance in the allogeneic setting might be due to enhanced cytotoxic T cell activity in the absence of Gal-1, while Gal-1 derived from NK cells was sufficient to induce apoptosis in activated T cells [42]. A tolerogenic role of Gal-1 has also been described in the context of bacterial infections [43] and in *T. cruzi* infected mice, in which Gal-1 is produced and secreted by B cells [44]. While Gal-1 is dispensable for normal development, at least in mice, it has important functions in modulating innate and adaptive immune mechanisms.

To further investigate the impact of Gal-1 on neuroblastoma aggressiveness, we crossbred TH-MYCN mice to Gal-1^−/−^ mice. We demonstrated that the Gal-1 gene dosage did not affect tumour incidence, growth rate, or survival probability, but was correlated with alterations in the immune phenotype exemplified by a reduction of CD4^+^ T cell infiltration in tumours of Gal-1^−/−^ mice [45]. Tumour infiltration by macrophages and NK cells was not affected by Gal-1 gene dosage in TH-MYCN mice. These findings are in line with the previously described role of Gal-1 in the tumour immune escape [46]. Here, Gal-1 was not only linked to induction of apoptosis in activated T cells [47] requiring tumour-immune cell contact [48] but also to induction of regulatory T cells [49]. Additionally, Gal-1 released by endothelial cells can induce apoptosis of activated but not resting T cells as a direct consequence of Gal-1 binding [47]. Hence, these findings confirm that T cells are main effectors of Gal-1 mediated immunomodulation. In addition to transgenic mouse models syngeneic transplantation models present one step further towards in vivo studies and are widely accepted as in vivo cell culture models. In the absence of a conditional Gal-1 ko mouse model, syngenic transplantation helped to dissect the effects of tumor-derived and host-derived Gal-1. Splenocytes from A/J mice receiving syngeneic NXS2 neuroblastoma cells with down-regulated Gal-1 (Gal-1 low) secreted higher amounts of IFN-γ and displayed enhanced cytotoxic T-cell function compared to NXS2 control cells or NXS2 cells overexpressing Gal-1 (Gal-1 high) [50]. Consequently, supernatants of NXS2 Gal-1 high or NXS2 control cells suppressed dendritic cell (DC) maturation and induced T cell apoptosis, while DCs and T cells exposed to supernatants from NXS2 Gal-1 low cells were largely unaffected. Moreover, shRNA-mediated downregulation of Gal-1 in murine cell lines leads to a significant decrease of tumour growth when transplanted subcutaneously into immunocompetent mice. [45]. Remarkably, low Gal-1 expressing murine NB cells also induced significantly lower numbers of liver metastases. Thus, Gal-1 levels affect aggressiveness and immune responses in both, transplanted and genetic models or murine neuroblastoma.

In addition to the altered immune phenotype of TH-MYCN/Gal-1^−/−^ mice, these animals suffer from splenomegalies indicated by a significantly higher spleen weight observed in tumour bearing Gal-1^−/−^ mice. These splenomegalies could be explained by a lower migratory capacity of Gal-1 deficient CD4^+^ T cells, while proliferation and apoptosis of CD4^+^ T cells was unaltered [45]. TH-MYCN/Gal-1^−/−^ mice present with higher infiltration of CD11^+^ dendritic cells compared to Gal-1^+/−^ or Gal-1 wt animals suggesting an enhanced tumour antigen presentation as a response to Gal-1 knock-out. However, tumours derived from mice lacking Gal-1 showed a reduction in CD31 endothelial cell staining confirming a role for Gal-1 in tumour angiogenesis depending on the Gal-1 gene dosage [45]. These findings are in line with a previously described role of Gal-1 in tumour angiogenesis, since Gal-1 was found to be overexpressed in both, tumour cells and tumour-associated endothelial cells, respectively [20]. It has been shown that Gal-1 enhances angiogenesis by interacting with N-glycans on the surface of endothelial cells in the extracellular space, and by augmenting VEGF signalling [27]. Furthermore, blocking Gal-1 functions using the small molecular inhibitor OTX008 reduced tumour growth and angiogenesis by targeting VEGFR-2 expression [51].

Standard therapies for aggressive neuroblastoma include surgery as well as chemo- and radiotherapy. Response to the latter therapeutic modalities is strongly modulated by the tumour microenvironment and development of resistance with subsequent disease relapse is frequently observed in aggressive type 2 neuroblastoma [52]. As both angiogenesis and the immune response have a strong impact on the outcome of radiotherapy in cancer, it remains an important task to understand the role of modifying factors, including Gal-1.

## 4. Gal-1 Expression and Its Impact on Radiotherapy

For neuroblastoma, standard of care includes surgery, chemotherapy and also radiotherapy. Since Gal-1 is overexpressed in the chemo- and radioresistant subtype of neuroblastoma, it can be considered as a potential therapeutic target for neuroblastoma therapy. Still, tumour irradiation is mainly performed by indirectly ionizing electromagnetic photons generated by an X- or γ-ray source, while only specialised centres make use of charged particles including protons, which are directly ionizing. Radiotherapeutic approaches mainly aim to induce DNA damage to kill malignant cells, as unrepaired DNA double-strand breaks subsequently induce apoptosis, mitotic catastrophes or autophagy. Thus, DNA damage repair pathways play a crucial role in determining the clinical outcome of radiotherapy, rendering it essential to find predictive biomarkers and novel targets for radiotherapy [53,54]. Gal-1 is induced by low dose ionizing radiation (0.5 Gy) in the tumour vasculature as well as in tumour-associated endothelial cells. Furthermore, specific enrichment of Gal-1 on the surface of tumour cells and stromal cells in the tumour microenvironment in vivo could be demonstrated using an isotope-labeled peptide, Anginex, which specifically binds to and inhibits Gal-1 functions [20]. Furthermore, co-cultivation of tumour cells and endothelial cells enhanced expression of Galectin-1 upon ionizing radiation, which might protect the tumour from radiation-induced cell death. It has been described that clinical or even subclinical doses sufficient to induce Gal-1 in the tumour microenvironment could be used to target Gal-1 by Anginex-loaded particles containing additional drugs [55]. Besides its role in direct induction of cell death as a consequence of unrepaired DNA damage and DNA double strand breaks, radiation also causes abscopal effects, defined as an action at a distance from the irradiated volume but within the same organism [56]. These systemic responses might be a consequence of radiation-induced release of immunogenic factors via a process termed “immunogenic cell death” (ICD). While several mediators of these abscopal effects have been identified, including pro-inflammatory cytokines, infrequency of these effects have been attributed to counterbalancing recruitment of myeloid-derived suppressor cells, enrichment of regulatory T cells and increased TGFβ-levels (reviewed in [57]). It is tempting to speculate that the immune-suppressive functions of Gal-1 also help tumours to dampen immune responses upon ionizing radiation. This hypothesis could be tested e.g., in the TH-MYCN model, in which differences in T cells and antigen-presenting cells between tumours with or without functional Gal-1 could be analysed. Hence, Gal-1 blockade might be beneficial in combination with radiation therapy by increasing immune responses towards the tumour.

Additionally, Gal-1 is not only an important player in the tumour microenvironment by modifying the immune response and angiogenesis, but it is also affected by tumour hypoxia. Several reports indicate a positive feedback loop expression between Hif1α, the major transcription factor involved in adaptation to hypoxic condition, and Gal-1 via the H-RAS oncogenic pathway [28]. Gal-1 expression is thus induced as a consequence of oxygen limitation in hypoxic environments. Hypoxic conditions limit the efficacy of radiotherapy as the formation of DNA-damaging H_2_O_2_ hydroxyl radicals requires oxygen. Thus, hypoxia within tumours causes a three-fold increase in the radiation dose required to generate the same amount of DNA damage when compared to normoxic conditions [28]. In addition to hypoxia, Gal-1 expression is induced by ionizing radiation of different cancer cells including breast, cervical and glioma cells [28,58]. Our own preliminary data point to Gal-1 upregulation on mRNA and protein level in Kelly and SY5Y human neuroblastoma cell lines upon ionizing radiation. However, shRNA mediated downregulation of Gal-1 in murine neuroblastoma cell lines did not alter the tumour cell responses to radiation with respect to clonogenic growth and cell cycle distribution. These findings confirm our previous results, which indicate a paracrine rather than an autocrine role for Gal-1 in neuroblastoma. Upon ionizing radiation, cells undergo autophagy or apoptosis as a consequence of DNA double-strand and single-strand breaks [28]. While it remains to be determined if Gal-1 mechanistically contributes to radiation responses, it can be hypothesized that upregulation of Gal-1 expression in a hypoxic tumour microenvironment additionally helps to stifle the immune responses to radiation.

## 5. Conclusions

Gal-1 is a multifaceted protein regulating different aspects of tumour biology. While necessary for balancing immune responses and angiogenic processes in physiological settings, tumours exploit these functions to escape from attacks of the host immune systems and to better cope with hypoxic conditions. Several strategies are currently evaluated to identify the best method to interfere with Gal-1 functions in therapeutic settings. One promising approach is to combine radiotherapy and Gal-1 blockade with the aim to reactivate immune responses towards the tumour and to limit angiogenic responses. Combining immune checkpoint inhibitors with blocking Gal-1 functions might also be an option to boost immunogenic anti-tumour responses. On the other hand, radiation-induced upregulation of Gal-1 could be used to selectively deliver drugs to Gal-1 positive cells in the tumour microenvironment. The results of these studies are anticipated to inform us about the optimal use of Gal-1 directed strategies in tumour therapy.
